# A comparison of phenotypic variation in *Triticum durum* Desf. genotypes deposited in gene banks based on the shape and color descriptors of kernels in a digital image analysis

**DOI:** 10.1371/journal.pone.0259413

**Published:** 2022-02-17

**Authors:** Elżbieta Suchowilska, Marian Wiwart, Urszula Wachowska, Wioleta Radawiec, Maciej Combrzyński, Dariusz Gontarz

**Affiliations:** 1 Department of Genetics, Plant Breeding and Bioresource Engineering, University of Warmia and Mazury in Olsztyn, Olsztyn, Poland; 2 Department of Entomology, Phytopathology and Molecular Diagnostics, University of Warmia and Mazury in Olsztyn, Olsztyn, Poland; 3 Department of Thermal Technology and Food Process Engineering, University of Life Sciences in Lublin, Lublin, Poland; 4 Polskie Zakłady Zbożowe Lubella GMW Ltd. LP, Lublin, Poland; KGUT: Graduate University of Advanced Technology, ISLAMIC REPUBLIC OF IRAN

## Abstract

Durum wheat is the tenth most valuable crop on a global scale. The aim of this study was to compare the phenotypic variation of *T*. *durum* accessions of different origin with contemporary spring cultivars of this cereal species. One hundred and two accessions and 12 contemporary cultivars of *Triticum durum* Desf. as well as Kamut® wheat (*T*. *turanicum*), a *Triticum* species closely related to *T*. *durum*, were analyzed. The aim of this study was to describe the degree of variation in the phenotypic traits of grain and selected traits associated with technological quality. The examined genotypes were characterized by considerable phenotypic variation, and they can be a valuable source of material for genetic recombination in durum wheat breeding. The analyzed accessions were characterized by a higher average content of protein (16.48 vs. 14.56%) and wet gluten (38.04 vs. 32.07%), higher Zeleny sedimentation values (69.7 vs. 60.4ml), and higher flour strength (W index values of 404.64 vs. 353.47) than the reference cultivars. The kernels of the evaluated accessions and cultivars did not differ significantly in average crease depth, but significant differences were observed in the values of descriptors directly linked with kernel size, especially kernel image area and minimal Feret diameter. The traits responsible for the processing suitability of grain were more strongly correlated with color descriptors than shape descriptors, which suggests that color parameters can be used to select high-quality breeding material. The analyzed accessions have two major weaknesses, namely relatively low yields (22.6 dt ha^-1^ on average) and undesirable grain color, indicative of low carotenoid concentration. The accessions deposited in gene banks do not meet the relevant agronomic requirements. However, both grain yield and carotenoid concentration are polygenic traits which can be improved if desirable combinations of QTLs are assembled in breeding lines and cultivars.

## Introduction

In recent years, the global area under *Triticum durum* Desf., the most economically important tetraploid wheat species, was estimated at 13 million ha, and annual yields reached nearly 40 million tons of grain [1]. Durum wheat is generally better adapted to high temperatures and semiarid climates than bread wheat [2]. Due to climate change, the growing region of durum wheat in Europe has expanded to include the central part of the European continent. Several EU countries, including Germany, Austria and Slovakia, have developed new durum wheat cultivars that are characterized by high processing suitability of grain and are well adapted to the climatic and agronomic conditions of Central Europe [3–5].

Rapid progress in durum wheat breeding has increased yields and reduced the protein content of grain. An increase in gluten strength and dough toughness has markedly improved the quality of pasta, but the processing suitability of contemporary durum wheat cultivars continues to decline [6]. The technological quality of grain is a polygenic trait that encompasses various parameters, mostly protein content, the content and quality of gluten, and the concentrations of carotenoids, in particular lutein and zeaxanthin, which are responsible for the desired color of grain and pasta [7]. Durum wheat has similar nutritional value and health benefits to bread wheat [8]. Milling value is a very important parameter in grain processing, and it is influenced by various factors, mostly grain vitreousness and the proportion of the starchy endosperm in total kernel weight. Starchy endosperm cells are packed with insoluble storage components, mainly starch and protein, which account for more than 80% of grain mass [9]. An increase in the ratio of kernel surface area to kernel volume increases the proportion of the pericarp and seed coat and decreases flour yield. Cultivars that produce rounded grain with a spherical shape and a shallow crease are most desirable in the milling industry. Roundness is indicative of grain maturity and high grain filling, associated with high flour yield. In comparison with bread wheat, the grain of durum wheat and other tetraploid wheat species is more elongated, which can compromise its milling value [10, 11]. The optimal varieties for *T*. *durum* breeding by genetic recombination should be characterized by high yield potential, as well as desirable phenotypic traits of grain such as high milling value and high processing suitability of semolina. Digital image analysis of grain shape and color supports rapid and effective selection of source material.

The possibilities offered by digital image analysis in research on cereal grain have been recognized already in the late 1980s [12]. Digital image analysis has been used to identify wheat cultivars [13, 14], evaluate grain contamination with seeds of other plant species [15] and assess the prevalence of infections caused by pathogens of the genus *Fusarium* [16]. Six wheat species (*T*. *aestivum*, *T*. *spelta*, *T*. *polonicum*, *T*. *dicoccon*, *T*. *durum* and *T*. *monococcum*) were discriminated by digital image analysis based on eight shape descriptors and six color descriptors in *HSI* (Hue, Saturation and Intensity) and L*a*b (Luminance, a* and b*) models [17]. Our previous study revealed that digital image analyses of kernel shape and color are highly useful for discriminating bread wheat and spelt hybrids and their parental forms [18]. Other researchers have demonstrated that kernels of diploid einkorn and ancient tetraploid emmer varieties adjust to the lens and that the curvature values at kernel poles are superior to those of modern “bread” varieties. Kernels of modern varieties (hexaploid common wheat) have an ellipsoidal shape with an aspect ratio of 1.6, whereas varieties of tetraploid durum, Polish wheat and hexaploid spelt resemble ellipsoids with an aspect ratio of 2.4 [19]. Digital image analysis has been also used in studies of durum wheat. Already 25 years ago, Symons et al. [20] relied on this technique to count specks in semolina. They analyzed 22 grain samples representing different locations and genotypes and found that digital image analysis generated by far more reliable results than visual counting. Novaro et al. [21] applied digital image analysis to predict semolina yields in 327 grain samples from 20 and 22 *T*. *durum* cultivars grown in various regions of Italy. Shape descriptors that were most strongly correlated with semolina yields were identified in a multiple regression analysis. The Fluoroscan F2000 system [22] significantly facilitates analyses of cereal species. The system supports fast and accurate speck analysis in flour products, such as semolina, rye, wheat, oats and barley, as well as measurements of aleurone, bran and ash particles in the flour industry. In a study investigating the vitreousness of durum wheat grain, Wang et al. [23] analyzed grain images with the GrainCheck 310 instrument (FOSS). The results of image analyses were processed with an artificial neural network, and the authors concluded that the GrainCheck 310 machine was characterized by superior performance relative to human inspectors. The EyeFoss™ (FOSS) image analyzer also relies on an artificial neural network to objectively evaluate the quality of bread wheat, durum wheat and barley grain. The machine can assess 10,000 kernels in just four minutes [24]. In recent years, numerous attempts have been made to use hyperspectral reflectance imaging in image analysis. Chen et al. [25] relied on this technique to analyze typical defects on the surface of durum wheat kernels. The analysis was conducted within a wavelength range of 40–1000 nm with the neighboring bands 2.73 nm apart. Based on the selected bands, 710 black germ kernels, 627 break kernels and 1,169 healthy kernels were classified with the highest discrimination accuracy of 95.6%, 96.7% and 98.5%, respectively. In turn, Vermeulen et al. [26] attempted to discriminate between common wheat and durum wheat kernels based on the results of infrared hyperspectral imaging, morphological criteria, protein content and the ratio of vitreous/non-vitreous kernels. By combining image-based morphometric characteristics with near-infrared hyperspectral imaging, the authors were able to detect fraud in sample classification with 99% accuracy.

The selection of source material is a very important consideration in creative plant breeding. Regardless of the applied breeding method, initial material is always the source of the most desirable genes. The milling value of durum wheat is largely influenced by the morphological and anatomical traits of grain. Therefore, the aim of this study was to compare the phenotypic variation of *T*. *durum* accessions deposited in gene banks with contemporary spring cultivars of this cereal species. The shape and color of whole kernels were evaluated in a digital image analysis, and the shape of their horizontal cross-sections was analyzed.

## Materials and methods

### Materials

The experimental material comprised 102 spring accessions and 12 cultivars of *T*. *durum* and Kamut® wheat (Table 1). The accessions were obtained from the National Plant Germplasm System, USA (Cltr and PI), and the seeds of durum wheat cultivars were acquired from the Crop Research Institute in Prague, Czech Republic (CZ), and Lubella Food Ltd. LP in Lublin, Poland (LU).

**Table 1:** Accessions and cultivars of *T*. *durum* and Kamut® wheat examined in the study.

Nº	Accession	Nº	Accession	Nº	Accession	Nº	Accession/cultivar
1	Cltr 10135	30	Cltr 15070	59	Cltr 2800	88	PI 585011
2	Cltr 10136	31	Cltr 15095	60	Cltr 3137	89	PI 585013
3	Cltr 11476	32	Cltr 15119	61	Cltr 3138	90	PI 585020
4	Cltr 11776	33	Cltr 15137	62	Cltr 3140	91	PI 585021
5	Cltr 11880	34	Cltr 15139	63	Cltr 3243	92	PI 585023
6	Cltr 11943	35	Cltr 15147	64	Cltr 3251	93	PI 585199
7	Cltr 12062	36	Cltr 15159	65	Cltr 7666	94	PI 585201
8	Cltr 12066	37	Cltr 15169	66	Cltr 7680	95	PI 585205
9	Cltr 12067	38	Cltr 15280	67	Cltr 7681	96	PI 7653
10	Cltr 12452	39	Cltr 15326	68	Cltr 7789	97	PI 7792
11	Cltr 12616	40	Cltr 15386	69	PI 8076	98	PI 8373
12	Cltr 12621	41	Cltr 15450	70	PI 8164	99	PI 8629
13	Cltr 12920	42	Cltr 15482	71	PI 8214	100	PI 585203
14	Cltr 12924	43	Cltr 15493	72	PI 10207	101	PI 585207
15	Cltr 13135	44	Cltr 15892	73	PI 13854	102	PI 591761
16	Cltr 13245	45	Cltr 15895	74	PI 13855	103	Atoudur(CZ)
17	Cltr 13246	46	Cltr 17057	75	PI 31940	104	Floridou(CZ)
18	Cltr 13337	47	Cltr 15019	76	PI 40938	105	Durafox(CZ)
19	Cltr 13918	48	Cltr 15096	77	PI 41027	106	Stelladur(CZ)
20	Cltr 14041	49	Cltr 15439	78	PI 41028	107	Duramant(1)
21	Cltr 14593	50	Cltr 12063	79	PI 42115	108	Duramont(LU)
22	Cltr 14597	51	Cltr 15509	80	PI 584832	109	IS Duragold(2)
23	Cltr 14701	52	Cltr 17058	81	PI 584833	110	Tamadur (3)
24	Cltr 1471	53	Cltr 17240	82	PI 584834	111	Floradur (3)
25	Cltr 14761	54	Cltr 17291	83	PI 584835	112	Duralis(1)
26	Cltr 14965	55	Cltr 17339	84	PI 584836	113	IS Duranegra (2)
27	Cltr 14978	56	Cltr 17406	85	PI 584840	114	Durasol (4)
28	Cltr 14979	57	Cltr 17747	86	PI 585009	115	Kamut®
29	Cltr 15024	58	Cltr 2793	87	PI 585010		

The accessions marked with the letters “Cltr” and “PI” were obtained from the National Plant Germplasm System (USA). The seeds of durum wheat cultivars were acquired from the Crop Research Institute in Prague, Czech Republic (CZ), and Lubella in Lublin, Poland (LU). (1)–Saaten-Union, Germany, (2)–Istropol Solary a.s., Slovakia, (3)- Probstdorfer Saatzucht Austria, (4)—Dr. Berthold Alter, Germany

### Field experiment

Grain was obtained from a field experiment conducted at the Agricultural Experiment Station in Bałcyny near Ostróda, Poland (53°36’N, 19°51’E). Durum wheat was grown on Cambisol soil suitable for wheat cultivation. The experiment was conducted using a randomized complete block design with three replications. The plot area was 9 m^2^. All genotypes were sown at the same density (220 kernels/m^2^). NPK fertilizer was applied at a pre-sowing rate of 18/60/90 kg ha^−1^, and a supplemental rate of 60 kg N ha^−1^ was applied at the end of the stem elongation stage (BBCH 47–52) [27]. Weeds were controlled with the herbicide Mustang 305 SE (Dow AgroSciences, Poland) at 0.5 l/ha. Fungicides were not applied. Grain for the experiment was harvested in the fully ripe stage (BBCH 92). Grain yield per ha was estimated based on grain yield per plot.

### Analysis of traits related to the technological quality of grain

The moisture content of kernels (%), one kernel weight–OKW (mg), kernel thickness (mm) and hardness were determined with the Single Kernel Characterization System (SKCS 4100, Perten). One grain sample consisted of 300 kernels. The concentrations of basic nutrients in whole kernels were determined with the Infratec 1241 (FOSS, Denmark) instrument. The following parameters were analyzed: crude protein content on a dry matter basis, wet gluten content, Zeleny sedimentation value, bulk density, and the W index (gluten strength). All measurements were performed in duplicate for each biological replicate. The instrument was calibrated according to the instructions provided by FOSS Poland.

### Analysis of kernel cross-sections

**Fig 1:**
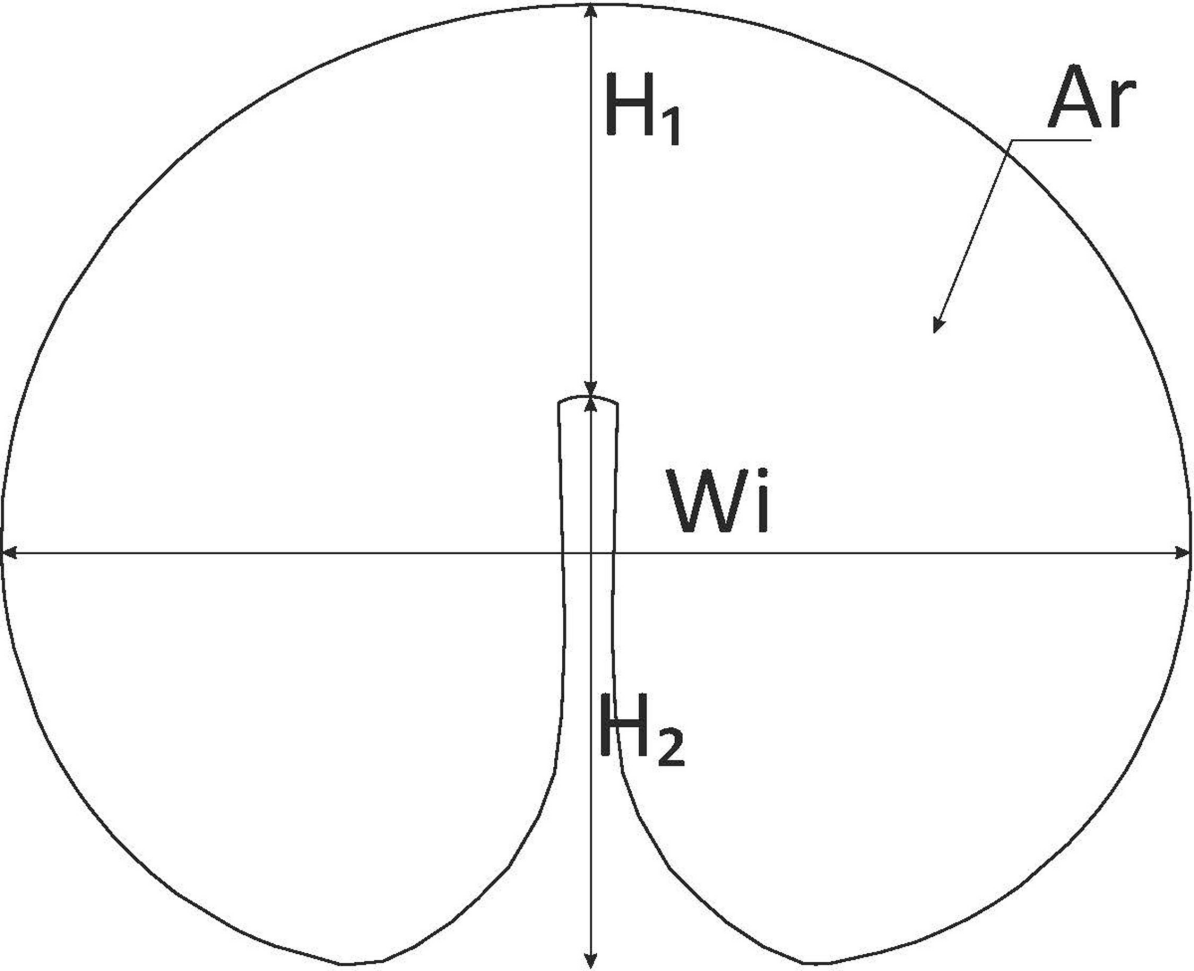
Diagram of the horizontal cross-section of a kernel and shape descriptors measured in microscopic images. Ar–total cross-sectional area, Wi–total cross-sectional width perpendicular to the crease, H_1_ –distance from bottom of the crease to kernel edge, H_2_ –crease depth.

The horizontal cross-sections of kernels were observed under the Tagarno Trend digital microscope (Tagarno, Denmark). Kernels were dissected manually with a scalpel in the central part, perpendicular to the crease, and four shape descriptors were measured: total cross-sectional area (A), total cross-sectional width (W), distance from the bottom of the crease to kernel edge (H_1_), and crease depth (H_2_) (Fig 1).

### Digital image analysis

Kernels were subjected to digital image analysis with the use of a method similar to that described by Goriewa-Duba et al. [17]. Digital images were acquired with a flatbed CCD scanner (Epson Perfection V370 Photo, Epson, Shinjuku, Tokyo, Japan). The image analysis was performed in three replications in the ImageJ program (v. 1.52r) [28]. Each replication consisted of 100 randomly selected kernels that were placed on the scanner screen with the crease down. A dark paper background with the predominance of the blue component (*R* = 100, *G* = 140, *B* = 200) was used. 24-bit color images with 300 dpi resolution were saved in BMP format. A median filter was applied (radius of five pixels, one replication) at the beginning of image segmentation. The color thresholding procedure was performed in an identical manner for all analyzed images, and a lower threshold value was set for the color component R at 130. In these procedures, 24-bit RGB images are thresholded based on hue, saturation, intensity (*HSI*), as well as red, green and blue components in the *RGB* color model, and the values L*a*b* in the CIELAB color space.

The following shape descriptors represented by individual blobs (regions of interest, ROI) were determined in images of individual kernels: area (A) (mm^2^), perimeter (PE) (mm), circularity (CI), Feret diameter (FD), minimal Feret diameter (MFD), aspect ratio (AR), roundness (RO), and solidity (SO) [17] (S1).

For color analyses, 24-bit color images were converted to three 8-bit images in channels *R*, *G* and *B* (S2). The color analysis was conducted based on the average values of variables *RGB* for every ROI, which were later used to calculate the values of *HSI* and L*a*b*. Parameter *H* denotes hue, *S* denotes saturation, *I* denotes intensity, L* denotes luminance, a* denotes redness–greenness, and b* denotes yellowness–blueness. Parameters *R*, *G* and *B* were converted to *H*, *S* and *I* and to L*, a* and b* with the use of the formulas proposed by Wiwart et al. [29].

### Statistical analysis

The results were processed statistically using Statistica 13.3 software [30]. The significance of differences between mean values was estimated by analysis of variance, and mean values were compared in Tukey’s multiple comparison test at *p<*0.01. All results were processed by correlation analysis. The values of the parameters measured in all accessions and cultivars were subjected to agglomerative hierarchical clustering (Ward’s method with the application of Euclidean distances) and principal component analysis (PCA).

## Results

### Traits related to the technological quality of grain

**Fig 2:**
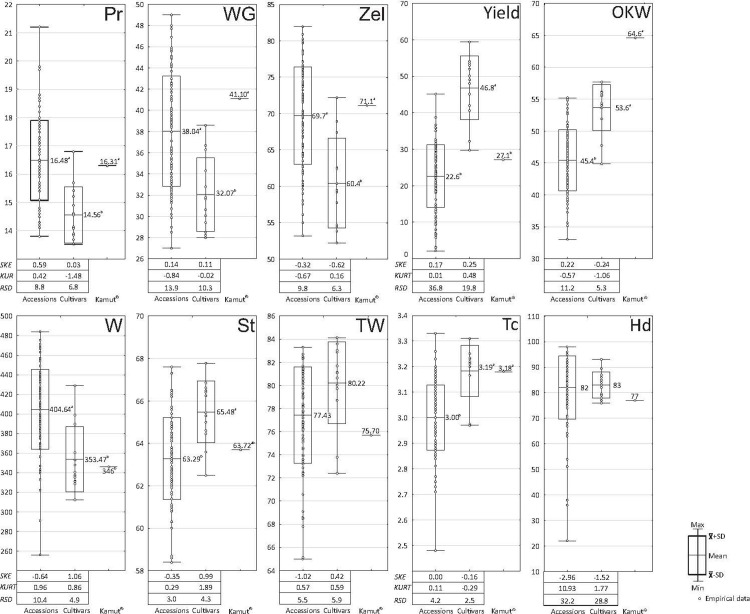
Protein content (Pr), wet gluten content (WG), starch content (St), Zeleny sedimentation value (Zel), W index (W), test weight (TW), yield (Yield), one kernel weight (OKW), hardness score (Hd) and thickness (Tc) of kernels in 102 accessions and 12 cultivars of T. durum and Kamut® wheat. SKE–skewness, KURT–kurtosis, RSD -relative standard deviation (%).

The values of nine traits related to grain quality and grain yield in 102 *T*. *durum* accessions, 12 cultivars of durum wheat and Kamut® wheat are presented in Fig 2. Significant differences in the average values of five traits determined with the Infratec® instrument (protein content, wet gluten content, starch content, Zeleny sedimentation value, flour strength) were noted between the studied cultivars and accessions. The test weight was the only parameter where significant differences were not observed. The average protein and gluten content, Zeleny sedimentation value and the W index were significantly higher in durum wheat accessions than in the reference cultivars, but they did not differ significantly from the values noted in Kamut® wheat (Fig 2). Interestingly, flour strength (W index) was significantly lowest in Kamut® wheat (346). Protein content is the most important indicator of the processing suitability of grain. The average protein content was 13% higher in the analyzed accessions than in the reference cultivars. The highest protein content (16.8%) was determined in cv. Atoudur, and it was considerably higher than the average value for all accessions. Significantly higher average gluten content, and higher values of Zeleny sedimentation and the W index indicate that the accessions deposited in gene banks are generally characterized by high processing suitability of grain and can be used in breeding practice to develop new cultivars of high technological quality. Significant differences in test weight were not determined between the evaluated genotypes, but the average test weight was higher in cultivars (80.22 kg) than in accessions (77.43 kg) and Kamut® wheat (75.70 kg). These findings indicate that the grain of the evaluated accessions was less well filled, and in this respect, the studied accessions ranked between Kamut® wheat and the reference cultivars. The frequency distribution of the discussed parameters was approximately normal, as indicated by the absolute values of skewness and kurtosis that exceed 1 only in several cases. The average value of OKW was significantly lower in the evaluated accessions (45.4 mg) than in cultivars (53.6 mg) and Kamut® wheat (64.6 mg). The average grain yield (estimated based on the average plot yield) was significantly highest in the reference cultivars (46.8 dt ha^-1^) relative to durum wheat accessions (22.6 dt ha^-1^) and Kamut® wheat (27.1 dt ha^-1^). The performance of the highest-yielding accession No. 30 (39.0 dt ha^-1^) was below the average yield of all cultivars. Grain yield was characterized by considerable variation, as demonstrated by high values of relative standard deviation (RSD) which reached 36.8% in accessions and 19.8% in cultivars. The examined cultivars and accessions did not differ significantly in the average hardness score, but this parameter varied considerably (RSD of 32.2% and 28.8% for accessions and cultivars, respectively), and kurtosis values were high (10.93 and 1.77, respectively). High kurtosis is indicative of heavy-tailed distribution, and data tend to be concentrated around the average value. The average kernel thickness, a parameter that is indirectly linked with grain plumpness, was similar in the studied cultivars (3.19 mm) and Kamut® wheat (3.18 mm), and it was significantly lowest in accessions (3.00 mm).

**Fig 3:**
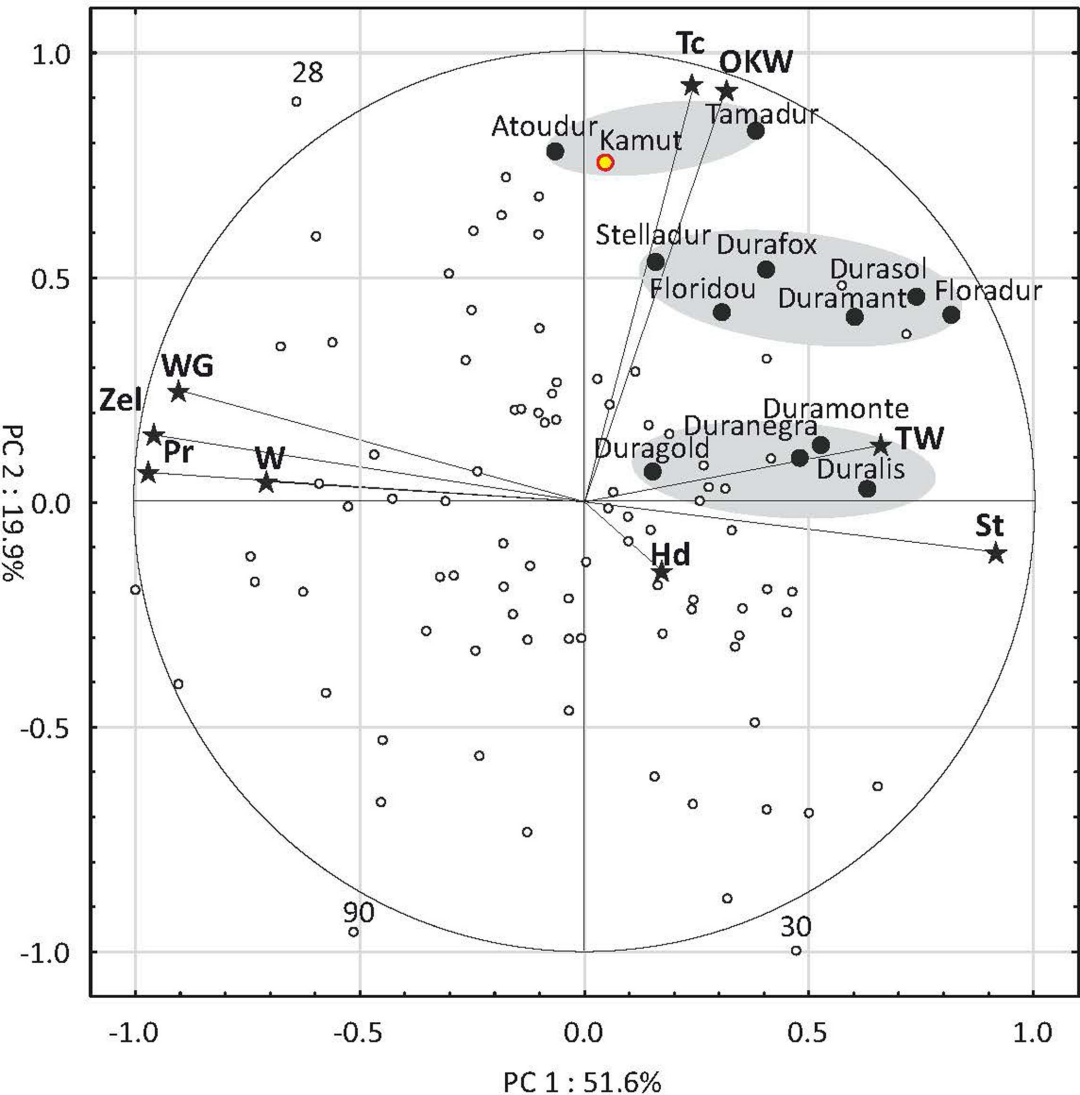
PCA results for traits related to the technological quality of 102 accessions and 12 cultivars of T. durum as well as Kamut® wheat. Pr—protein, TW—test weight, WG—wet gluten, St–starch, W–W index, Zel–Zeleny sedimentation number, OKW–one kernel weight, Tc–thickness, Hd—hardness score.

The results of the Principal Component Analysis (PCA) for nine traits, excluding yield, are presented in Fig 3. The circles in the biplots have a radius of 1 which represents the maximum absolute value of Pearson’s correlation coefficient between the variable (carotenoid) and the principal component. Vectors denote the direction and strength of the correlations. The first two principal components (PC1 and PC2) explained 71.5% of total variance. The hardness score (Hd) followed by the W index were characterized by the lowest discriminatory power (Fig 3). The points corresponding to the reference cultivars were distributed in three clearly separated regions. The first region was composed of cvs. Atoudur and Tamadur as well as Kamut® wheat with high values of OKW and Tc. The second region contained cvs. Duramonte, Duranegra, Duragold and Duralis with high values of TW and high starch content. The grain of the above cultivars was also characterized by low values of traits that are directly related to the processing suitability of flour/semolina, namely protein content, W index, Zeleny sedimentation value, and wet gluten content. The third region was situated in between the above regions, and it was composed of cvs. Stelladur, Durafox, Floradur, Floridou, Durasol and Duramant. Only eight accessions (7.8%) were identified in the above regions. The regions characteristic of specific accessions were difficult to identify because their discriminatory power was relatively low. The vast majority of the identified accessions were grouped in the central and bottom part of the diagram. The accessions located on the left side of the diagram were characterized by higher protein content, higher gluten content and higher gluten quality than the reference cultivars. The PCA revealed very high variation in the studied accessions as well as cultivars. The values of traits that are directly linked with yield potential (OKW, S and Tc) were high in the reference cultivars. Many of the studied accessions were characterized by higher protein content, protein quality and grain hardness in comparison with the reference cultivars.

Three confidence ellipses were identified. The first ellipse contains cvs. Atoudur and Tamadur and Kamut® wheat; the second ellipse contains cvs. Floradur, Durasol, Duramant, Floridou, Durafox and Stelladur; and the third ellipse contains cvs. Duranegra, Duralis, Duragold and Duramonte. Numerical values denote the most outlying accessions.

### Analysis of kernel cross-sections

**Fig 4:**
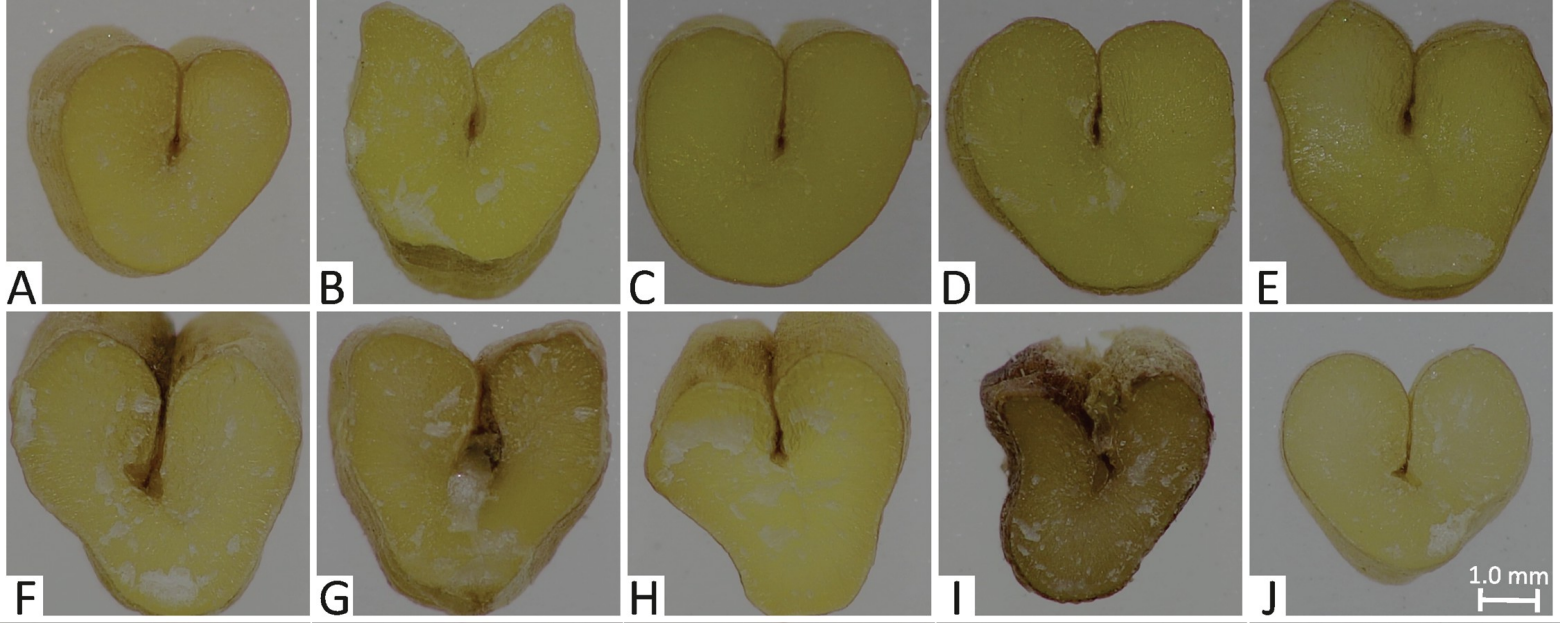
Horizontal cross-sections of kernels of selected T. durum cultivars and accessions. A—Duralis, B—Kamut®, C—Floradur, D—Durasol, E—Duragold, F—Cltr 12924, G—Cltr 14761, H—Cltr 7680, I—PI 8164, J—PI 41028.

In Fig 4, the variability in the experimental material is presented in exemplary microscopic images of 10 durum wheat cultivars and accessions.

**Fig 5:**
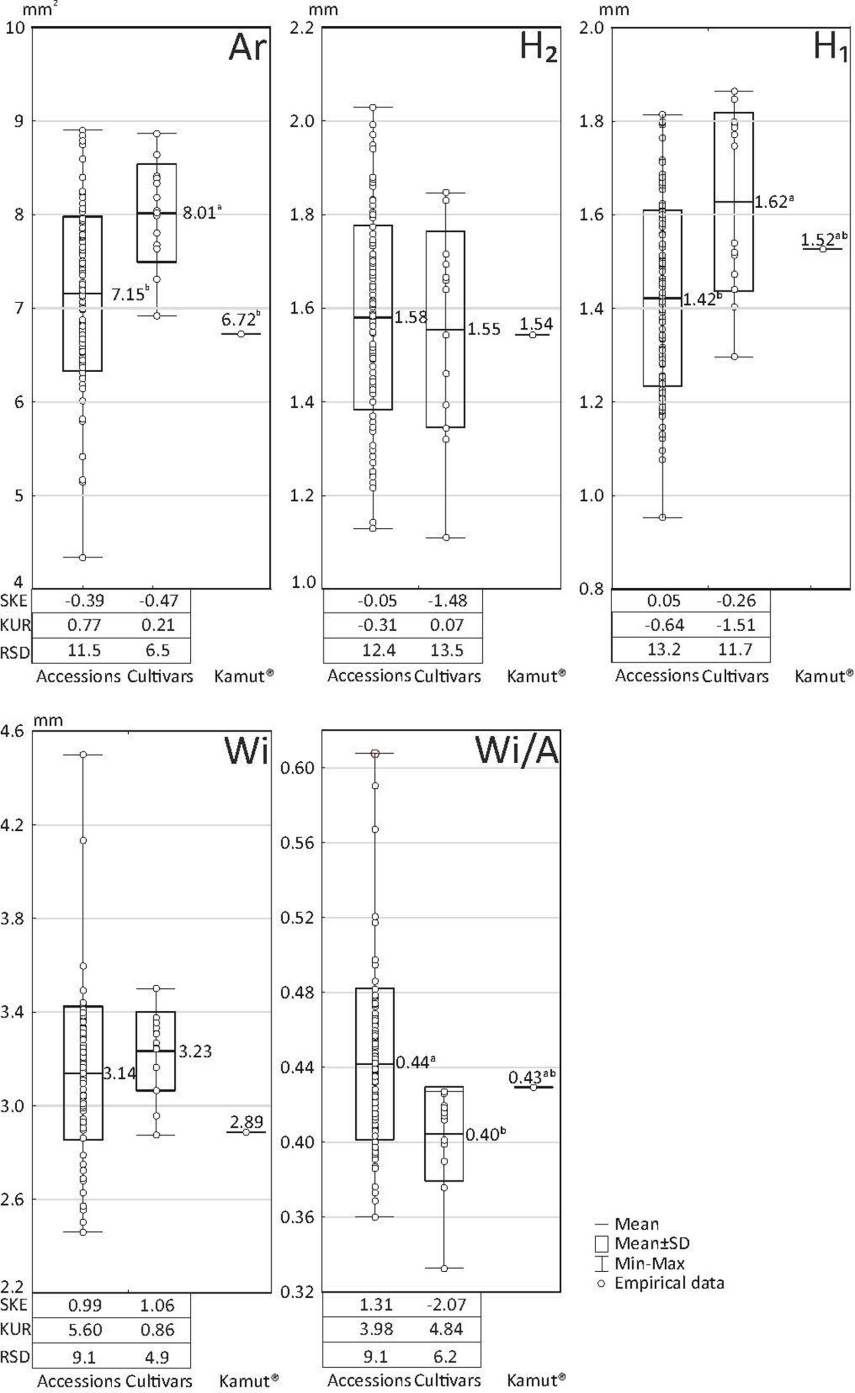
Variation in four descriptors of the cross-sectional images of kernels of 102 accessions and 12 cultivars of *T*. *durum* as well as Kamut® wheat. Ar–total cross-sectional area, H_2_ –crease depth, H_1_ –distance from the bottom of the crease to kernel edge, Wi–total cross-sectional width (refer to Fig 1).

The variation in the descriptors of the cross-sectional images of kernels of the studied accessions and cultivars of *T*. *durum* and Kamut® wheat is shown in Fig 5.

**Fig 6:**
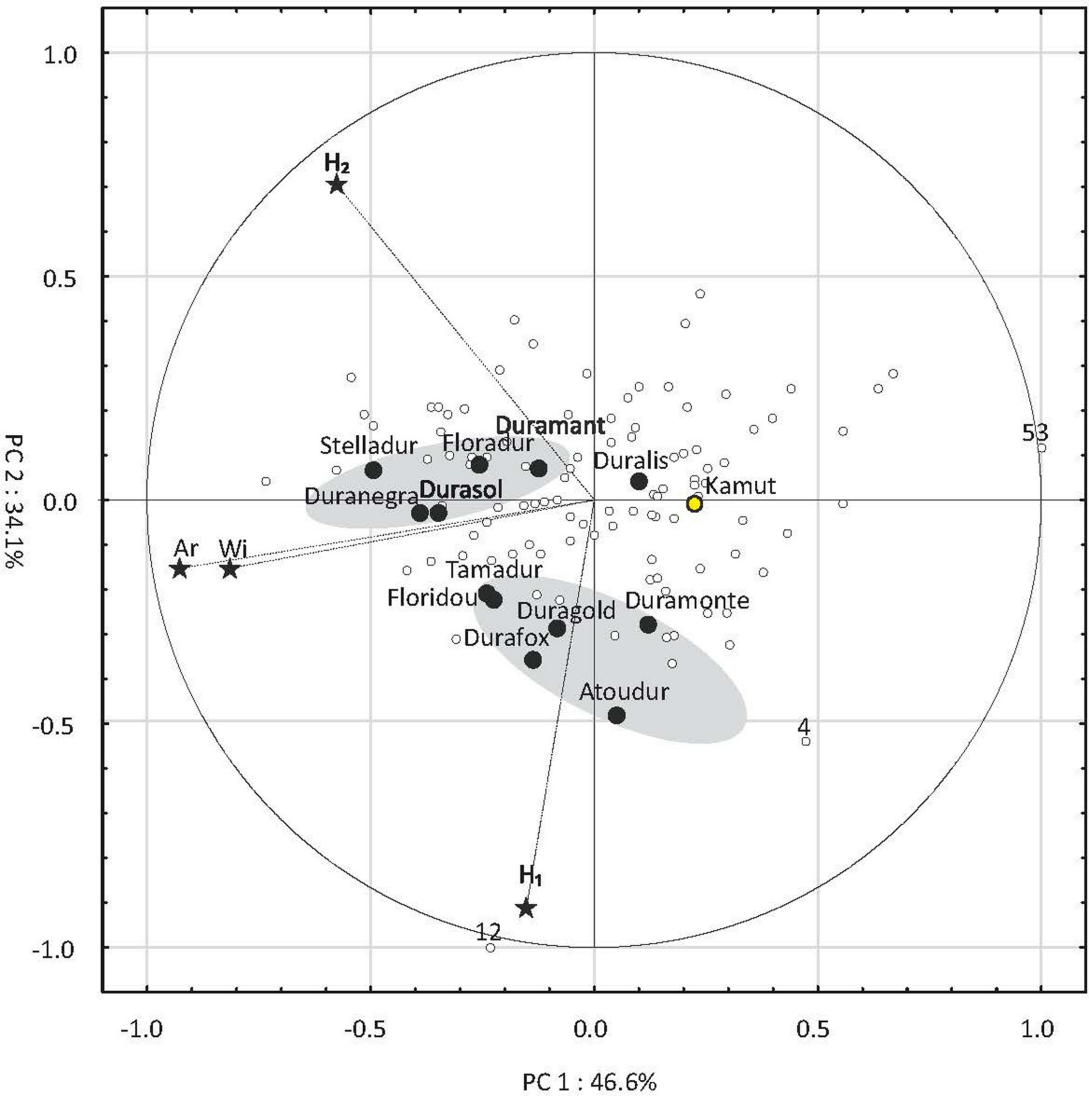
PCA results for the descriptors of the cross-sectional images of kernels of 102 accessions and 12 cultivars of *T*. *durum* as well as Kamut® wheat. Ar–total cross-sectional area, H_1_ –distance from the bottom of the crease to kernel edge, H_2_ –crease depth, Wi–total cross-sectional width perpendicular to the crease. Two confidence ellipses were identified for cvs. Duramant, Floradur, Duranegra, Durasol and Stelladur, and cvs. Tamadur, Floridou, Duragold, Duramonte, Durafox and Atoudur. Numerical values denote the most outlying accessions.

Significant differences were observed in the mean values of three variables: (1) cross-sectional area, which was significantly highest in cultivars (8.01 mm^2^) and somewhat higher in accessions (7.15 mm^2^) than in Kamut® wheat (6.72 mm^2^); (2) distance from the bottom of the crease to kernel edge; (3) ratio of cross-sectional width to cross-sectional area (Wi/A ratio). The latter parameter was significantly highest in accessions (0.44) and lowest in cultivars (0.40). The Wi/A ratio is determined by kernel flatness. With the exception of crease depth, RSD values were higher in accessions than in cultivars, ranging from 9.1% to 13.2%. The analyzed parameters differed also in skewness and kurtosis. Excluding cross-sectional width, skewness was lower in cultivars than in accessions, which suggests that distribution was left-skewed and that most values were above the average in the group of cultivars. In turn, higher skewness values for cross-sectional width in the group of cultivars points to a higher proportion of genotypes with below-average values of Wi relative to accessions. Kurtosis values were particularly high for cross-sectional width (5.60 in accessions) and the Wi/A ratio (3.98 in accessions and 4.82 in cultivars). High positive values indicate that the empirical values were not dispersed and tended to concentrate around the mean. Interestingly, the ratio of cross-sectional height (H_1_+H_2_) to cross-sectional width (Wi) was highly similar in cultivars (0.98) and accessions (0.97), and only somewhat higher in Kamut® wheat (1.06). The PCA results for variables Ar, H_2_, H_1_ and Wi are presented in Fig 6. The Wi/A ratio was not considered in PCA because this variable is directly correlated with parameters Ar and Wi. All four variables had high and similar discriminatory power, as demonstrated by the values of the corresponding vectors that approximated 1. The first two principal components (PC1 and PC2) explained more than 80% of total variance, which indicates that they had high discriminatory power. The points corresponding to the analyzed cultivars formed two characteristic clusters. The first cluster featured cvs. Duramant, Stelladur, Floradur, Duranegra and Durasol, and the second cluster contained cvs. Tamadur, Floridou, Duragold, Duramonte, Durafox and Atoudur. Cultivar Duralis was highly similar to Kamut® wheat.

The points corresponding to accessions were distributed mainly in the right and central part of the biplot, which is indicative of low values of Ar and Wi. Approximately 39% of accession points were located inside or in the close proximity of cultivar clusters, which suggests that these accessions were highly similar to cultivars in terms of the analyzed parameters.

### Digital image analysis

**Shape analysis.** The values of shape descriptors in the images of wheat kernels are presented in Table 2.

**Table 2:** The values of shape descriptors in the images of kernels of the analyzed accessions and cultivars of *T*. *durum* and Kamut® wheat.

		A (mm^2^)	PE (mm)	CI	FD (mm)	MFD (mm)	AR	RO	SO
Accessions (n = 102)	Mean	18.79^c^	17.92^b^	0.73^a^	7.22^b^	3.33^b^	2.20^b^	0.97^a^	0.46
	RSD %	9.6	5.5	4.3	6.7	5.3	8.4	0.4	7.8
	Min-Max	14.00–25.68	15.84–22.55	0.60–0.81	6.07–9.55	2.59–3.69	1.81–3.00	0.96–0.98	0.34–0.56
Cultivars (n = 12)	Mean	19.91^b^	18.37^b^	0.74^a^	7.36^b^	3.46^a^	2.16^b^	0.97^a^	0.47
	RSD %	8.3	4.6	2.3	5.1	4.2	4.7	0.1	4.6
	Min-Max	16.24–22.60	16.63–19.87	0.72–0.76	6.66–8.01	3.12–3.63	2.03–2.32	0.97–0.98	0.44–0.50
Kamut®		25.67^a^	22.54^a^	0.63^b^	9.54^a^	3.38^ab^	2.86^a^	0.35^b^	0.97

A—area, PE—perimeter, CI—circularity, FD—Feret diameter, MFD—minimal Feret diameter,

AR—aspect ratio, RO—roundness, SO–solidity; ^a,b,c^- mean values marked with the same letter do not differ significantly in Tukey’s multiple comparison test at p < 0.01.

**Fig 7:**
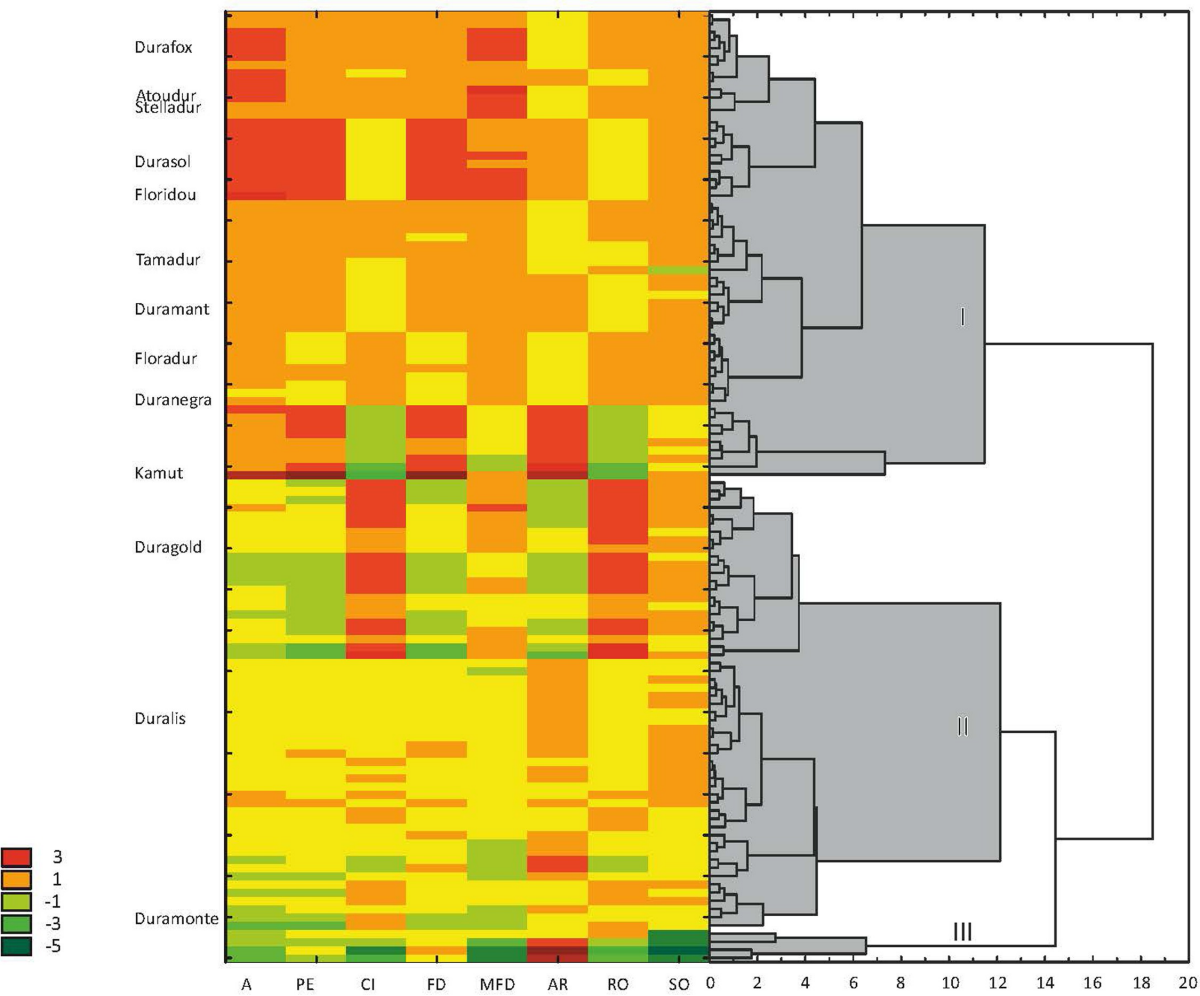
A heat map and the results of cluster analysis of kernel shape descriptors in 102 accessions and 13 cultivars of *T*. *durum* and in Kamut® wheat (marked on the left side of the map). A—area, PE—perimeter, CI–circularity, FD—Feret diameter, MFD—minimal Feret diameter, AR—aspect ratio, RO—roundness, SO solidity. I, II, III–major clusters.

In the group of eight variables, SO was the only parameter that did not differ significantly between the studied cultivars and accessions of durum wheat and Kamut® wheat. Shape descriptors were characterized by relatively low variation within both groups of genotypes, and RSD values ranged from 0.1% for RO in cultivars to 9.6% for A in accessions. A comparison of the mean values of shape descriptors revealed lower average values of A (by nearly 6%) and MFD (by nearly 4%) in accessions than in cultivars. Circularity values were somewhat lower, and AR values were higher, which suggests that the grain of durum wheat accessions was generally smaller and more elongated that the grain of cultivars. The results of hierarchical cluster analysis of eight shape descriptors are presented in Fig 7, and they were used to identify three main clusters.

Cluster I consisted of 47 accessions and 10 cultivars characterized by large kernel area and perimeter, as well as high values of FD and MFD. The low values of CI and RO indicate that these kernels had an elongated shape. Cluster II contained three cultivars (Duragold, Duralis and Duramonte) and 51 accessions that formed two large groups. The first group was composed of genotypes with rounded kernels (high values of CI and RO) and low values of AR and FD. The second group was characterized by relatively low values of MFD and above-average values of AR, which indicates that these kernels had lower width values and were more elongated in comparison with the first group. The second group contained cvs. Duralis and Duramonte. Cluster III was composed of only four accessions with the most elongated and least regularly shaped kernels (lowest values of MFD and CI, high values of AR, and lowest values of SO).

### Color analysis

The color of kernel images was initially described in the 24-bit *RGB* model, and the resulting values were converted to *HSI* and L*a*b* color space [29]. The color descriptors for both models are presented in Table 3. Significant differences in the average values of all three variables were observed between *T*. *durum* cultivars and accessions. Hue values were lower in accessions, which suggests that their grain tended to be redder than the grain of cultivars (30.42 vs. 32.77), whereas higher values of *S* and *I* indicate that grain color was characterized by higher saturation and lightness. The mean values in L*a*b* color space also differed significantly between groups, and the greatest difference was noted in the value of b*. The average value of this descriptor was more than two units higher in cultivars than in accessions. Parameter b* describes the difference between yellow and blue color components. Cultivars were characterized by a higher positive value of b* than accessions, which points to a higher contribution of yellowness in their grain.

**Table 3:** The values of color descriptors in the images of kernels of the analyzed accessions and cultivars of *T*. *durum* and Kamut® wheat.

		*H*	*S*	*I*	L*	a*	b*
Accessions (n = 102)	Mean	30.42^b^	0.26^a^	0.55^a^	65.28^b^	-3.49^ab^	7.12^b^
	RSD %	4.9	2.7	3.6	0.2	7.7	16.6
	Min-max	24.62–33.04	0.25–0.29	0.51–0.61	64.80–65.54	-4.01–-2.72	3.52–9.08
Cultivars (n = 13)	Mean	32.77^a^	0.25^b^	0.50^b^	65.56^a^	-3.85^b^	9.16^a^
	RSD %	2.1	1.2	3.1	0.1	5.3	5.6
	Min-max	31.22–33.72	0.25–0.26	0.51–0.56	65.44–65.66	-4.16–-3.44	8.07–9.86
Kamut®		31.14^ab^	0.25^b^	0.53^ab^	65.42^ab^	-3.42^a^	8.55^ab^

*H–*hue, *S–*saturatio*n*, *I–*intensity, L^*^–luminance, a*- red/green, b*- yellow/blue; ^a,b,^- mean values marked with the same letter do not differ significantly in Tukey’s test at p < 0.01.

**Fig 8:**
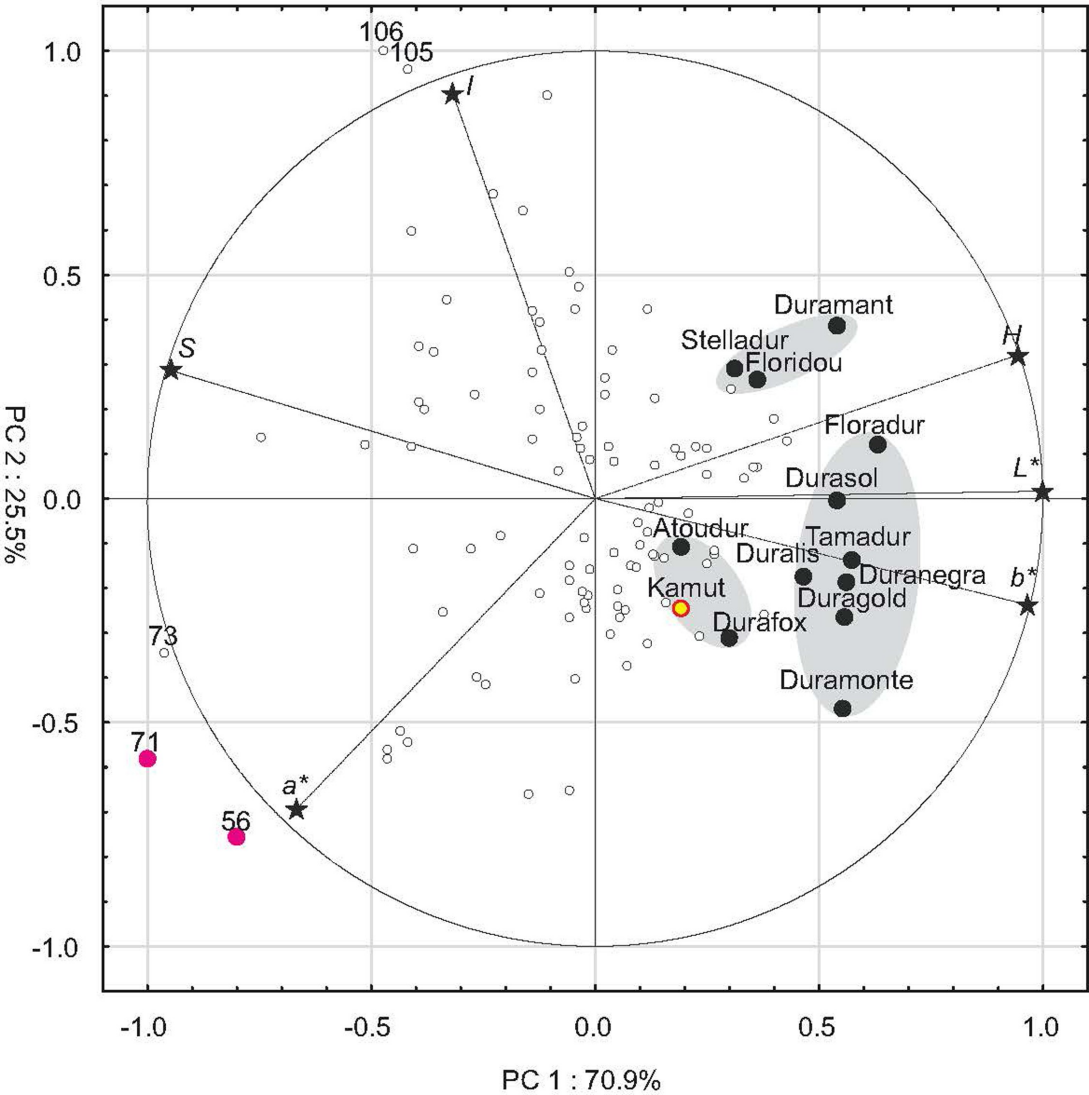
PCA results for kernel color descriptors in 102 accessions and 12 cultivars of *T*. *durum* and Kamut® wheat. H—hue, S–saturation, I—intensity, L*- luminance, a*—redness–greenness, b*—yellowness–blueness. Three confidence ellipses were identified. The first ellipse contains cvs. Duramant, Floridou and Stelladur; the second ellipse contains cvs. Floradur, Durasol, Tamadur Duranegra, Duralis, Duragold and Duramonte, and the third ellipse contains cvs. Atoudur and Durafox and Kamut® wheat. Numerical values represent the most outlying accessions. Numbers 56 and 71 denote accessions with purple grain.

All of the analyzed genotypes were strongly discriminated by the six color descriptors in PCA (Fig 8). The first two PCs explained 96.4% of total variance, and all six descriptors had high discriminatory power. All reference cultivars were grouped in three clusters on the right side of the biplot, which indicates that the color of their grain was generally characterized by higher values of *H*, L* and b* in comparison with accessions. The first cluster contained cvs. Duramant, Stelladur and Floridou with low values of a* and high values of *H* denoting the lowest contribution of redness in pericarp color.

The second group was composed of cvs. Floradur, Durasol, Tamadur, Duralis, Duranega, Duragold and Duramonte with high values of b* and L* and low values of *S*. In these cultivars, the pericarp was characterized by a lighter and less saturated color with a higher contribution of yellowness relative to the first group. The third group featured cvs. Atoudur and Durafox and Kamut® wheat whose color was similar to that noted in the second group, but these kernels were characterized by higher color saturation and lower values of L*, which implies that they were somewhat darker and more intense in color. The points representing most accessions were grouped in the central part of the biplot, with very few outliers. Very high values of *I* were noted in six accessions (their grain was lightest in color of all analyzed genotypes), whereas the highest values of a* and the lowest values of *H* were observed in accessions No. 56 and 71 with purple-colored grain. In these accessions, grain color was characterized by the highest contribution of redness.

Significant (*p<0*.05) values of the Pearson’s correlation coefficient *r* for traits related to the technological quality and yield of *T*. *durum* accessions and reference cultivars are presented in Table 4. These parameters were most strongly correlated with protein content, but negative values of *r* were noted for grain yield and traits that were most closely related to yield potential (from -0.913 for starch content to -0.214 for TKW). As expected, protein content was bound by strong positive correlations with the Zeleny sedimentation value and wet gluten content. Relatively weak correlations were noted between thickness and the remaining parameters (only thickness vs. OKW and yield at 0.837 and 0.408, respectively), and negative correlations were noted between kernel hardness vs. protein content, wet gluten content and the Zeleny sedimentation value (-0.298, -0.338 and -0.255, respectively).

**Table 4:** Statistically significant values of the Pearson’s correlation coefficient *r* for the traits related to the technological quality and yield of the analyzed *T*. *durum* accessions and reference cultivars.

	OKW	Protein	Test weight	Wet gluten	Starch	W index	Zeleny	Thickness	Hardness
Protein	-0.251*								
Test weight	0.293**	-0.649**							
Wet gluten		0.895**	-0.495**						
Starch		-0.912**	0.606**	-0.800**					
W index		0.627**		0.621**	-0.572**				
Zeleny		0.943**	-0.508**	0.917**	-0.855**	0.749**			
Thickness	0.837**								
Hardness		-0.298**	0.276*	-0.338**			-0.255*		
Yield	0.514**	-0.595**	0.584**	-0.496**	0.468**	-0.241*	-0.540**	0.408**	0.206*

*, **—the values of the Pearson’s correlation coefficient *r* significant at *p<* 0.05 and 0.01, respectively

The absolute values of *r* for seed hardness were not high, but they were statistically significant. Significant values of the correlation coefficient *r* for all analyzed image descriptors, the phenotypic traits of grain, and yield per unit area are presented in Table 5. Most image descriptors were significantly correlated with TKW, test weight, thickness and yield.

It should be noted that protein content, wet gluten content and the Zeleny sedimentation value were correlated mostly with color descriptors. The values of *r* were generally below 0.5, but they were statistically significant. The descriptors of the cross-sectional images of kernels were correlated mostly with OKW, thickness and, to a smaller extent, with test weight. In this case, the strongest correlations were noted between cross-sectional area vs. OKW and thickness (*r* = 0.637 and 0.602, respectively). Both traits were most strongly correlated with shape descriptors A and MFD (*r* = 0.600 to 0.819). Interestingly, hardness was not significantly correlated with any of the image descriptors. The absence of correlations between hardness vs. OKW, starch content and thickness suggests that *T*. *durum* genotypes can be selected for grain hardness independently of grain yield, color and kernel size.

**Table 5:** Statistically significant values of the correlation coefficient *r* for all image descriptors, the phenotypic traits of grain and yield per unit area.

	OKW	Protein	Test weight	Wet gluten	Starch	W index	Zeleny	Thickness	Hardness	Yield
Ar	0.637**	-0.221*	0.250*		0.228*			0.602**		0.382**
H_2_	0.210*									
H_1_	0.290**		0.201*					0.272*		0.214*
Wi	0.364**		0.232*					0.319**		0.207*
Wi/A	-0.408**	0.206*			-0.214*			-0.402**		
*H*	0.466**	-0.434**	0.381**	-0.265*	0.420**		-0.316**	0.374**		0.477**
*S*	-0.574**	0.409**	-0.501**		-0.368**		0.283**	-0.483**		-0.497**
*I*	-0.385**	0.339**	-0.620**	0.231*	-0.258*	0.229*	0.259*	-0.317**		-0.330**
L*	0.545**	-0.463**	0.465**	-0.272**	0.436**		-0.341**	0.447**		0.532**
a*	-0.292**	0.408**	-0.226*	0.340**	-0.420**		0.338**	-0.214*		-0.376**
b*	0.573**	-0.425**	0.498**	-0.211*	0.385**		-0.299**	0.480**		0.509**
A	0.819**							0.600**		0.313**
PE	0.697**							0.444**		
CI			0.349**							
FD	0.594**							0.327**		
MFD	0.782**							0.741**		0.446**
AR			-0.320**							
RO			0.291**							
SO	0.385**		0.319**			0.334**		0.273*		0.265*

Ar–total cross-sectional area, H_1_ –distance between the bottom of the crease and kernel edge, H_2_ –crease depth, W–total cross-section width perpendicular to the crease. Color descriptors in kernel image analysis: H—hue, S–saturation, I—intensity, L*—luminance, a*- redness–greenness, b*—yellowness–blueness. Shape descriptors in kernel image analysis: A—image area, PE—perimeter, CI–circularity, FD—Feret diameter, MFD—minimal Feret diameter, AR—aspect ratio, RO—roundness, SO–solidity; *, **—the values of the Pearson’s correlation coefficient *r* significant at *p<* 0.05 and 0.01, respectively

## Discussion

The genetic pool of modern crop cultivars continues to diminish, which is both the cause and effect of genetic erosion. Therefore, breeders search for new sources of genetic variation in three independent areas. The first approach relies on information about natural genetic variation in a given crop species, which is generally available in gene banks and can be used in breeding programs. In the second approach, genetically related taxa can be used as source materials in cross-breeding programs. Thirdly, genetic variation can be engineered through genetic transformation and induced mutagenesis. However, genetic engineering is fraught with numerous technical problems, and it is not widely approved by consumers [31], whereas induced mutagenesis in allopolyploid species is not highly effective [32]. Interspecific hybrids bred within the same genus or intergeneric hybrids can be difficult to develop due to genetic and physiological barriers. From the practical point of view, the approach that relies on natural genetic variation that is already present in a plant species appears to be most effective and least expensive. Contemporary cultivars are often closely related, and breeders frequently resort to sources of genetic variation that are stored in gene banks. However, detailed information is not available for many accessions, and the desired genetic traits may be difficult to identify. In this study, an attempt was made to determine the type and degree of phenotypic variation in the grain of selected spring accessions of durum wheat that can be potentially used for breeding new cultivars. One hundred and two accessions with the most desirable agronomic traits were selected from nearly 180 accessions that were reproduced in a field experiment. The selected genotypes were characterized by high resistance to pathogens, high yield, resistance to lodging and early maturation. In turn, the reference cultivars were characterized by high yield potential and desirable values of traits related to technological quality and processing suitability. Therefore, they constituted the optimal reference material.

An evaluation of grain yields was not the primary research objective. However, yield is a very important parameter and it should be noted that in the group of 102 *T*. *durum* accessions, the yield potential of 15.9% genotypes was within the range of values determined in the reference cultivars. Most of the studied accessions were characterized by higher average protein content (85% of accessions), higher wet gluten content (78% of accessions) and higher Zeleny sedimentation values (82% of accessions) in comparison with the reference cultivars. These attributes are typically bound by negative correlations with grain yield in most crop species, including wheat [33]. The above poses a considerable problem in breeding practice because high grain quality is difficult to reconcile with high yield per hectare. In this respect, the present findings indicate that the evaluated genotypes could constitute promising source material for quality breeding of *T*. *durum*.

Grain shape traits strongly influence yield and milling quality [34]. The dimensions and shape of wheat kernels are critical for grain processing and milling. Large spherical kernels are more desirable due to their higher milling value [35, 36]. Crease size influences milling value and flour yield because the presence of a crease complicates pearling [37]. A crease incision on the ventral side can promote the growth of fungal pathogens, including toxigenic fungi of the genus *Fusarium* [38]. Bread wheat and durum wheat cultivars with a shallow crease and, consequently, a low ratio of kernel surface area to kernel volume are most desirable. In this study, crease depth in most accessions was similar to that noted in contemporary cultivars, which suggests that these genotypes could be used for breeding new cultivars of durum wheat. The cross-sectional width of kernels in the analyzed accessions was similar to that observed in the reference cultivars, but these kernels were characterized by smaller cross-sectional area, lower values of H_1_ (distance from the bottom of the crease to kernel edge) and lower values of TW and TC, which indicates that the proportion of the starchy endosperm in total kernel volume was considerably lower. Lower values of the Wi/A ratio in the evaluated accessions indicate that their kernels were generally flatter than the kernels of the reference cultivars.

Artificial neural networks (ANN) and hyperspectral imaging (HI) techniques facilitate quick and automatic discrimination of the seeds of various crop species and cultivars [25, 39, 40]. As mentioned in the Introduction, these technologies are being implemented in industrial practice to develop new solutions. However, ANN and HI methods are used mainly to discriminate species rather than cultivars within a given species because greater differences in the morphometric characteristics of grain are generally observed between than within species. An evaluation of intraspecific differences requires detailed information about the type and degree of variation in shape and color descriptors which were analyzed in this study. Interestingly, the average values of most shape descriptors did not differ significantly between durum wheat accessions and reference cultivars. The only exceptions were A and MFD whose values were significantly lower in accessions than in cultivars. Both descriptors were positively correlated with OKW and yield. One kernel weight is associated with kernel volume which, in turn, is correlated with the cross-sectional area and cross-sectional width of kernels. Interestingly, CI was not correlated with any of the studied parameters. Circularity values range from 0 in extremely elongated objects (straight line) to 1 in an ideal circle. The average values of CI were highly similar in accessions and cultivars. Similarly to other tetraploid wheat species, *T*. *durum* has more elongated kernels than bread wheat, which is not a highly desirable trait in the milling industry [35]. The present experiment was conducted on the assumption that the grain of *T*. *durum* accessions would be more elongated that the grain of modern intensively-farmed cultivars. However, this assumption was not confirmed. Greater differences in color descriptors than shape descriptors were observed between the investigated accessions and cultivars. Grain color was analyzed in two models, *HSI* and L*a*b*. Color is described differently in each model [41], https://www.easyrgb.com/en/math.php), which is why all six descriptors were evaluated jointly in a multi-directional analysis, apart from ANOVA. The grain of the reference cultivars was characterized by higher values of b* and *H*, which points to a greater contribution of yellowness. The color of grain and end products is determined by phenotypic variation in grain pigments which is influenced by genetic factors, growing conditions and technological processes [7]. The color of pasta, determined by the concentrations of carotenoids (mainly lutein), is one of the most important indicators of the technological quality of durum wheat grain. For this reason, source materials with high carotenoid content are most desirable in breeding practice. However, high carotenoid content is a quantitative trait that is encoded by numerous QTLs on all chromosomes [7]; therefore, genotypes are difficult to select based on this trait. The results of PCA indicate that only a small number of accessions were characterized by desirable grain color and satisfactory carotenoid levels.

*Triticum turanicum*, a species closely related to *T*. *durum* and commercially known as Kamut®, was introduced to this study as a reference object. Kamut® is a trademark that has been used in marketing products of the protected cultivated *T*. *turanicum* variety QK-77 since 1990 when it was registered in the USA [42]. Kamut® wheat grain is highly suitable for the production of pasta and other products made from durum wheat, and it is characterized by high nutritional value and health benefits [43, 44]. It has low water requirements, which is an important consideration in an era of climate change [45]. In the current study, Kamut® wheat was more similar to accessions that to cultivars in terms of the average values of most traits related to grain quality. Kamut® wheat is a highly promising source material for cultivar breeding on account of its high protein content, high content of wet gluten, high gluten quality, as well as the highest TKW. Kamut® wheat was also more similar to the evaluated accessions than to cultivars in terms of shape descriptors in digital image analysis. The greatest differences were noted in the shape descriptors of whole kernels, which indicates that Kamut® wheat kernels were significantly largest and most elongated. Based on the average values of color descriptors, Kamut® wheat grain was ranked between cultivars and accessions. In terms of most parameters, Kamut® wheat grain was more similar to the grain of accessions than contemporary cultivars of *T*. *durum*.

Durum wheat is the tenth most valuable crop on a global scale. Most of the allelic variation of genes found in original wild relatives, which has gradually been lost through domestication and breeding, can be recovered only by going back to landraces [46].

## Conclusion

The analyzed collection materials of *T*. *durum* are characterized by considerable phenotypic variation, which indicates that selected genotypes could constitute valuable source material for genetic recombination. The traits responsible for the processing suitability and technological quality of grain were more strongly correlated with color descriptors than shape descriptors, which suggests that color descriptors can be effectively used to select high-quality breeding materials. The analyzed genotypes have two main weaknesses. The first is low yield, and the second is undesirable grain color, indicative of low carotenoid concentration. The accessions deposited in gene banks do not meet the relevant agronomic requirements, and they are unlikely to become direct precursors of valuable cultivars. However, both grain yield and carotenoid concentration are polygenic traits which can be improved if desirable combinations of QTLs are assembled in breeding lines and cultivars.

## Abbreviations

Ar: total cross-sectional area of the kernel
H_1_: distance between the bottom of the crease and kernel edge
H_2_: crease depth
Wi: total cross-section width perpendicular to the crease
*H*–hue: color descriptors in kernel image analysis
*S*: saturation
*I*: intensity
*L**: luminance
*a**: redness–greenness
*b**: yellowness–blueness
A–area: shape descriptors in kernel image analysis
PE: perimeter
CI: circularity
FD: Feret diameter
MFD: minimum Feret diameter
AR: aspect ratio
RO: roundness
SO: solidity
